# Time Series Analysis of Trends in Malaria Cases and Deaths at Hospitals and the Effect of Antimalarial Interventions, 2001–2011, Ethiopia

**DOI:** 10.1371/journal.pone.0106359

**Published:** 2014-11-18

**Authors:** Maru Aregawi, Michael Lynch, Worku Bekele, Henok Kebede, Daddi Jima, Hiwot Solomon Taffese, Meseret Aseffa Yenehun, Abraham Lilay, Ryan Williams, Madeleine Thomson, Fatoumata Nafo-Traore, Kesetebirhan Admasu, Tedros Adhanom Gebreyesus, Marc Coosemans

**Affiliations:** 1 World Health Organization, Global Malaria Program, Geneva, Switzerland; 2 World Health Organization, Country Office, Addis Ababa, Ethiopia; 3 Federal Ministry of Health, Addis Ababa, Ethiopia; 4 International Research Institute for Climate and Society (IRI), Earth Institute, Columbia University, New York, New York, United States of America; 5 Institute of Tropical Medicine, Antwerp, Belgium; Mahidol-Oxford Tropical Medicine Research Unit, Thailand

## Abstract

**Background:**

The Government of Ethiopia and its partners have deployed artemisinin-based combination therapies (ACT) since 2004 and long-lasting insecticidal nets (LLINs) since 2005. Malaria interventions and trends in malaria cases and deaths were assessed at hospitals in malaria transmission areas during 2001–2011.

**Methods:**

Regional LLINs distribution records were used to estimate the proportion of the population-at-risk protected by LLINs. Hospital records were reviewed to estimate ACT availability. Time-series analysis was applied to data from 41 hospitals in malaria risk areas to assess trends of malaria cases and deaths during pre-intervention (2001–2005) and post-interventions (2006–2011) periods.

**Findings:**

The proportion of the population-at-risk potentially protected by LLINs increased to 51% in 2011. The proportion of facilities with ACTs in stock exceeded 87% during 2006–2011. Among all ages, confirmed malaria cases in 2011 declined by 66% (95% confidence interval [CI], 44–79%) and SPR by 37% (CI, 20%–51%) compared to the level predicted by pre-intervention trends. In children under 5 years of age, malaria admissions and deaths fell by 81% (CI, 47%–94%) and 73% (CI, 48%–86%) respectively. Optimal breakpoint of the trendlines occurred between January and June 2006, consistent with the timing of malaria interventions. Over the same period, non-malaria cases and deaths either increased or remained unchanged, the number of malaria diagnostic tests performed reflected the decline in malaria cases, and rainfall remained at levels supportive of malaria transmission.

**Conclusions:**

Malaria cases and deaths in Ethiopian hospitals decreased substantially during 2006–2011 in conjunction with scale-up of malaria interventions. The decrease could not be accounted for by changes in hospital visits, malaria diagnostic testing or rainfall. However, given the history of variable malaria transmission in Ethiopia, more data would be required to exclude the possibility that the decrease is due to other factors.

## Background

**Figure 1:**
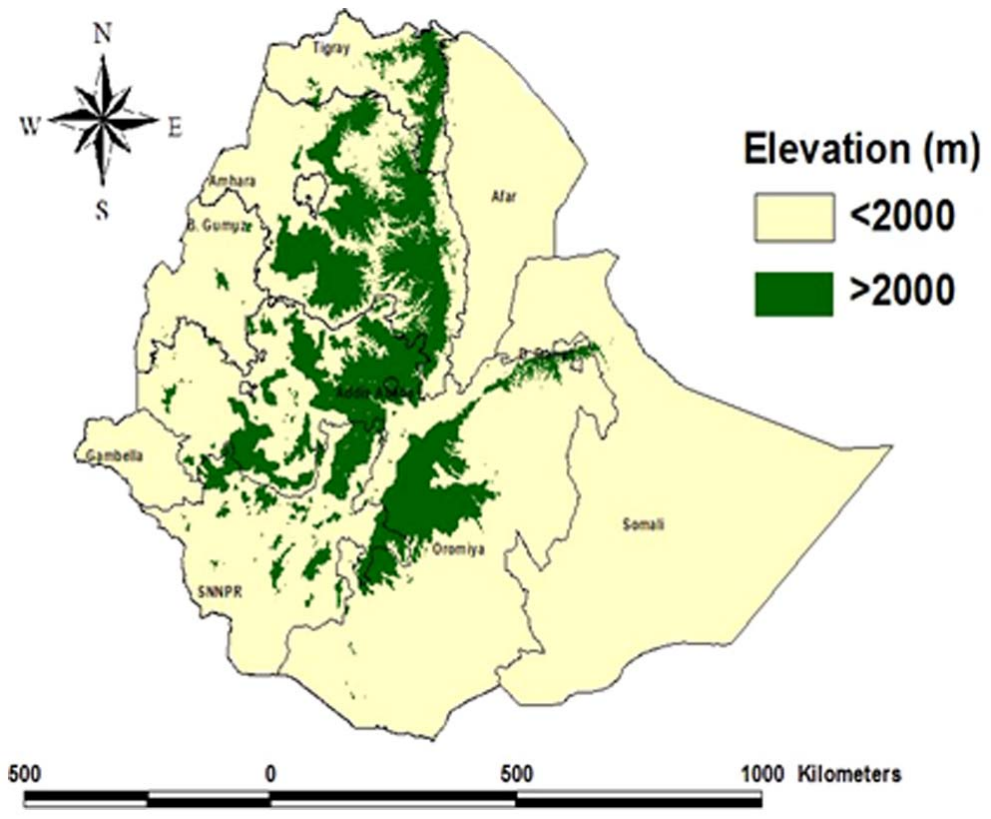
Administrative regions and areas below and above 2000 meters elevation in Ethiopia.

Malaria affects over 68% of the population in Ethiopia, a country of 94 million people in 2013 [1]. The disease is highly seasonal with varying intensity of transmission owing to altitudinal and climatic variations [2]. Across the nine administrative regions, areas that lie below 2000 m altitude are considered as malarious (Figure 1) [3]. The occurrence of malaria epidemics in the past indicates that there is little immunity in the majority of the population, owing to few infective mosquito bites per person per year [4], [5]. The high transmission season coincides with the cultivation months; hence malaria has a deleterious effect on agricultural production. *Plasmodium falciparum* accounted for nearly 55% of all malaria cases during 2008–2012 [6].

Malaria control has been one of the major components of the country's National Heath Sector Development and Poverty Reduction Strategy, put forth since 2004 in different phases, the most recent plan being for the period 2010–2015. With investment by the Global Fund to Fight HIV/AIDS, Tuberculosis and Malaria, the USA President's Malaria Initiative, World Bank and other development partners, the government has employed three key antimalarial interventions: i) distribution of long-lasting insecticidal nets (LLINs) through mass campaigns, to the entire population at risk; ii) indoor residual spraying (IRS) in designated epidemic-prone areas; and iii) increased diagnostic testing through rapid diagnostic tests (RDT) and microscopy, along with deployment of artemisinin-based combination therapy (ACT). Distribution of LLINs started in 2005. ACT was introduced to all public health facilities, free-of-charge to all age groups, as the first-line treatment for uncomplicated malaria in July 2004 [7].

Malaria control efforts have benefited from the national health sector development strategy which aims to increase access to effective health care by deploying health extension workers (HEWs) to provide integrated health promotion and treatment at peripheral health posts and at community level. By 2009 over 30,000 HEWs had been deployed. Treatment provided by HEWs targets the leading causes of death in children: malaria, pneumonia, and diarrhoea [8]. For malaria, the HEWs verify ownership and encourage use of LLINs; diagnose malaria with RDTs and treat confirmed cases; then refer suspected severe malaria cases to the health centres and hospitals.

This study aimed to (i) investigate the proportion of the population benefiting from malaria control interventions, (ii) assess trends in malaria cases and deaths in hospitals in malaria risk areas between 2001 and 2011, and (iii) examine the relationship between malaria interventions and the occurrence of malaria cases and deaths during 2001–2011. Attempts were made to take into account other factors which may affect malaria cases and deaths in hospitals and to employ statistical approaches focused on assessing changes in trends of malaria cases and deaths before and after the major scale up of malaria interventions since 2005. The results are discussed in light of international goals and targets for reducing malaria burden.

## Methods

### Ethical clearance

Ethical clearance was not required as the retrospective data used from the health facilities were monthly aggregates and anonymous counts of clinical cases and deaths.

### Intervention coverage

Information on malaria control interventions (LLINs, IRS, and ACTs) was obtained from national malaria control program (NMCP) records. As the programme started implementation of LLINs in 2005, the number of LLINs distributed to each region during 2005–2011 was recorded. The number of insecticide treated mosquito nets available before 2005 was assumed to be small. Regional population estimates were taken from the 2007 Ethiopian census and adjusted using the population growth of United Nations population estimates for all the study years [9]. The proportion of the population potentially protected by LLINs in a given year was calculated by assuming that each LLIN protected 1.8 persons and lasted three years (number of LLINs distributed during the 3 year period ×1.8/population). The proportion of the population protected with IRS nationwide was calculated by dividing the number of persons in households protected by IRS by the population at risk. The availability of ACT treatment was assessed by reviewing records of stock-outs at hospitals that were visited to access trends in malaria cases and deaths. ACTs were counted as available for a given month if ACTs were in stock for more than three weeks in a month.

### Malaria cases and deaths

Data on malaria outpatient cases, admissions and deaths were obtained from health facilities that provided inpatient services and were located below 2000 m or served catchment populations predominantly below 2000 m elevation (Figure 1). Of the total 120 inpatient facilities in Ethiopia, 62 facilities met the above criteria; 55 hospitals were visited, including three located in highland areas that predominantly serve populations from low land areas, while six hospitals were not visited for logistical reasons. Surveillance data were considered adequate if data on all indicators were available for more than 70% of months. Of 56 facilities visited, 41 facilities that had data for >70% of months between 2001–2011 were included in the final analysis.

At each visited hospital, monthly totals of outpatient visits, hospital admissions and deaths, and laboratory tests performed were reviewed. The total numbers of outpatient visits, admissions and deaths for all-causes and for malaria were recorded by two age groups (less than 5 years of age and 5 years old or greater). A suspected outpatient malaria case was defined as a visit in which malaria was the preliminary diagnosis in the outpatient record. A confirmed malaria case was defined if malaria parasites were demonstrated by microscopy (RDT results were not considered as they are used at the lower health facilities and community level by the HEWs). The slide positivity rate (SPR) was defined as the number of microscopy slides with malaria parasites divided by the number of microscopy slides reviewed. Laboratory records could not be broken down by age, nor could they be linked individually to outpatient or inpatient cases. Inpatient malaria cases and deaths were based on the diagnosis at discharge. The non-malaria cases and deaths, used for comparisons, were generated by subtracting malaria from the corresponding all-cause outpatient, admissions and deaths.

### Meteorological data

Enhanced National Climate Services (ENACTS) rainfall data that is quality controlled and blended satellite and observational data [10] from the National Meteorological Agency of Ethiopia was obtained by the International Research Institute for Climate and Society, Columbia University, New York, USA. These precipitation data (estimate of rainfall in mm) were aggregated for five major regions of the country and were used as the principal short-term predictor to assess the effect of climate on the trends of malaria cases and deaths [11].

### Statistical methods

Stata 11 was used to compile data by year and month and perform statistical analysis [12]. Trends in confirmed malaria cases and SPR, malaria inpatient cases, and malaria deaths were assessed by region. Malaria inpatient cases and malaria deaths were assessed by the two age groups. Confirmed malaria cases and SPR were assessed for all ages as these could not be disaggregated by age. Change in indicators over time was evaluated in three ways: i) by comparing the mean of annual values of cases and deaths during 2001–2005 (considered the pre-intervention period) to the observed value for 2011; ii) using a segmented regression model of an interrupted time series [13], comparing observed 2011 to predicted values in 2011, assuming a continuation of the pre-intervention time trend through 2006–2011; and iii) comparing the average for the observed values for 2006–2011 with the midpoint level predicted using segmented regression, assuming a continuation of the pre-intervention time trend through 2006–2011. The magnitude of changes in indicators was expressed as the relative percent change. For (ii) and (iii), the model adjusts the estimate for (1) possible time trend of the indicator during the pre-intervention period; (2) a possible immediate drop or rise of the indicator following the start of the intervention, and (3) a time trend on the indicator post-intervention. The 95% confidence intervals (CI) around the estimates were computed using the CIs around the regression coefficient estimates. A relative percent change in an indicator for which the CI does not include zero was considered statistically significant. In the time-series regression model for (ii) and iii), an Autoregressive Integrated Moving Average (ARIMA) model was used. A correlogram and partial autocorrelation plots were used to guide selection of the best ARIMA model [14].

To provide insight into whether changes in malaria cases and deaths were related to the introduction of malaria interventions in time, a sensitivity analysis was done by dividing the time series into two segments, a pre-intervention and post-intervention period, and varying the dividing point and evaluating the segmented regression model for each break point. The optimal breakpoint, or the month with the maximum change in the trend line, was considered to be the point where the amount of variation accounted for by the segmented regression model (R^2^) was greatest [15].

Month-to-month variability in indicators was evaluated using monthly values of the indicators during pre-and post-intervention periods using sinusoidal functions. To observe and remove short-term seasonal fluctuations, the monthly trends were extracted using a Hodrick-Prescott filter with monthly smoothing parameter [16]. Spectral analysis was done to detect the fundamental frequency components of the series. Additionally, month effects were parametrically modeled by using sinusoidal functions (Indirect Discrete Fourier Transform (IDFT).

The time trend in monthly rainfall, the principal short-term predictor of malaria burden [17], was plotted by region.

## Results

### Interventions

**Figure 2:**
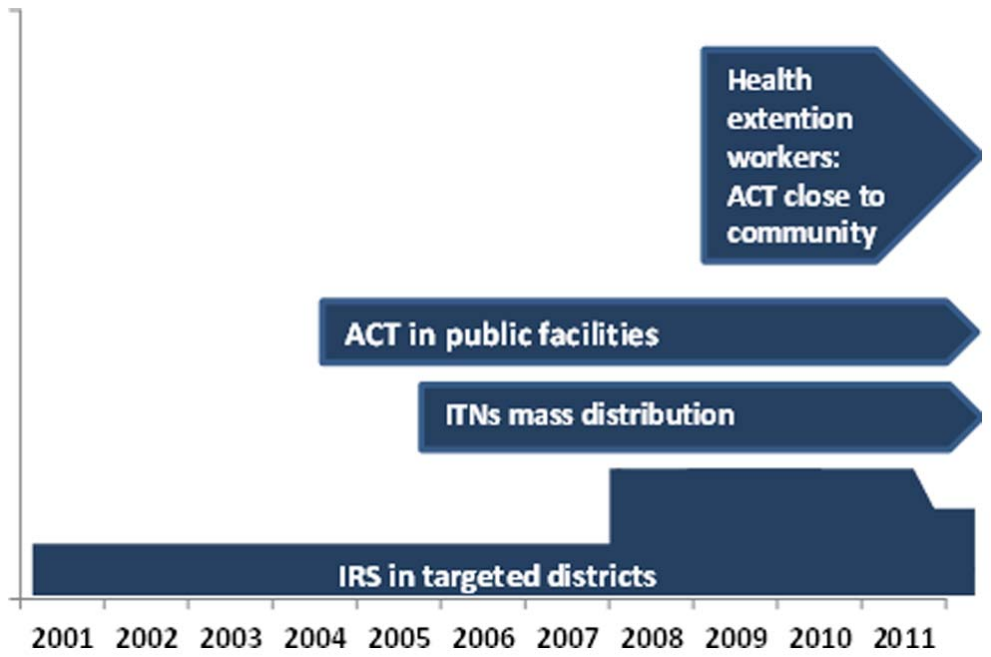
Timeline of implementation of antimalarial interventions, 2001–2011, Ethiopia.

The national malaria control programme distributed a total of 43.1 million LLINs during 2005–2011 targeting all populations in malarious areas, of which 23.8 million LLINs were distributed during 2005–2007, 2.1 million were distributed 2008–2009 and 17.1 million nets during 2010–2011 (Figure 2).

**Figure 3:**
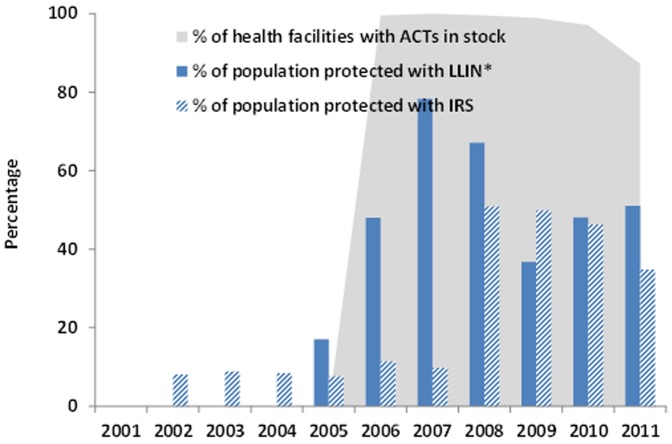
Percentage of population potentially protected with LLINs*, IRS and percentage of health facilities with stock of ACT available by year, 2001–2011, Ethiopia (*number of LLINs distributed during the 3 year period ×1.8/population).

The proportion of population potentially protected by LLINs increased from nearly zero in 2005 to 78% in 2007, and 51% in 2011 (Figure 3). The proportion of population protected with IRS increased from 10% in 2007 to 35% in 2011. In 40 hospitals, ACT stock records were complete for 80% of the months during 2006–2011 (no ACT stock records were available for 2004 and 2005). Stock out of ACT for any age group was very low during 2006–2011 and therefore availability of ACT was >99% during 2006–2009, 97% in 2010 and 87% in 2011. Over 50% of the stock outs in 2011 were in Oromia region.

### Yearly trends of malaria cases, admissions and deaths

**Figure 4:**
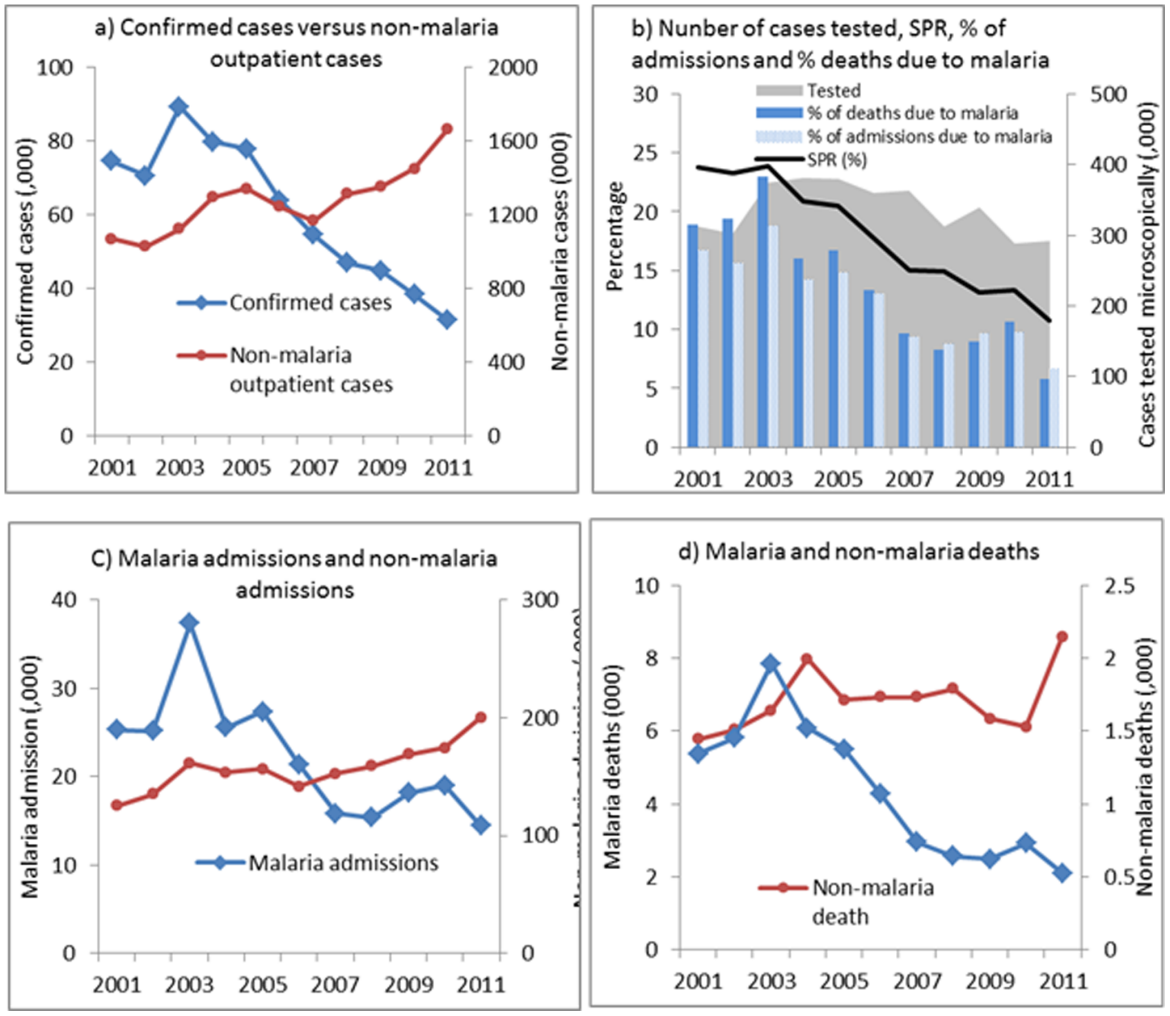
Trends of confirmed malaria cases and non-malaria outpatient visits (a); slide positivity rate and number of microscopic slides examined (b); malaria admissions and non-malaria admissions (c); malaria deaths and non-malaria deaths in all ages (d) in 41 hospitals, Ethiopia 2001–2011.

Of the sampled 62 hospitals, 55 hospitals were visited but only 49 hospitals met the criteria for collection of historical data. Data covering 2001–2011 was complete in 41 of the 49 hospitals which were the basis for the analysis in this study. The 8 hospitals with incomplete data comprised 4 from Oromia, 2 from Amhara and one each from Afar and Tigray. Confirmed malaria cases decreased from a mean of 78,325 during 2001–2005 to 30,780 cases in 2011 (Figure 4a), an observed decline of 61%, and a 66% decline (95% confidence interval [CI], 44–79%) from the number of cases predicted in 2011 if the trend during 2001–2005 had continued (Table 1). The number of confirmed cases at the mid-point of 2006–2011 was lower by 50% (CI 24–68%) than the values predicted. The SPR was 24% in 2001 and 11% in 2011 (Figure 4b); SPR decreased from a mean of 22% during 2001–2005 to 11% in 2011 (Table 1) and declined 37% (CI, 20%–51%) compared with the predicted value of 2011. Malaria inpatient cases and malaria deaths were highest in 2003, decreased during 2005–2008, and then rose slightly in 2009 but fell again in 2011 (Figure 4c, 4d). Overall, malaria inpatient cases in all ages were 54% lower and malaria deaths 68% lower in 2011 than that predicted by trends during 2001–2005, however, these declines were not statistically significant.

**Table 1:** Percentage change in malaria and non-malaria related indicators post-intervention years compared to pre-intervention period (2001–2005), by age, 41 hospitals <2000 m elevation, Ethiopia, 2001–2011.

Age group	Indicator	Indicator	Observed values and changes			Change (%) in predicted versus observed (95% CI)	
			Observed values in 2011	Preintervention ave (2001–2005)	Observed change in 2011 (vs ave 2001–2005)	Midpoint 2006–2011	2011
**Children <5 years**	**Malaria**	Outpatient malaria cases	21,257	32,287	−34	−47% (−70%–−6%)†	−57% (−78%–−15%)†
		Malaria admissions	3,998	6,773	−41	−80% (−92%–−51%)†	−81% (−94%–−47%)†
		Malaria deaths	119	289	−59	−64% (−80% – −37%)†	−73% (−86%–−48%)†
	**Nonmalaria**	Nonmalaria outpatient cases	175,028	121,849	44	−20% (−57%–47%)	−22% (−62%–59%)
		Nonmalaria inpatient cases	32,242	22,366	44	−3% (−29%–34%)	−3% (−33%–41%)
		Nonmalaria deaths	1,396	1,388	1	−28% (−42%–−11%)†	−40% (−53%–−23%)†
**5 years and above**	**Malaria**	Outpatient malaria cases	77,863	113,158	−31	−54% (−65%–−41%)†	−63% (−73%–−50%)†
		Malaria admissions	10,423	21,374	−51	−43% (−77%–41%)	−52% (−83%–39%)
		Malaria deaths	406	1,240	−67	−58% (−88%–46%)	−66% (−92%–46%)
	**Nonmalaria**	Nonmalaria outpatient cases	1,488,596	1,048,716	42	−15% (−39%–20%)	−10% (−39%–34%)
		Nonmalaria inpatient cases	167,603	123,765	35	−13% (−44%–33%)	−9% (−45%–51%)
		Nonmalaria deaths	7,192	5,249	37	−20% (−56%–47%)	−8% (−56%–89%)
**All ages**	**Malaria**	Outpatient malaria cases	99,121	145,445	−32	−53% (−65%–−36%)†	−62% (−73%–−45%)†
		Malaria admissions	14,421	28,147	−49	−45% (−77%–30%)	−54% (−83%–27%)
		Malaria deaths	525	1,530	−66	−59% (−86%–22%)	−68% (−91%–17%)
		Laboratory examined cases	292,801	350,086	−16	−33% (−51%–−7%)†	−46% (−63%–−21%)†
		Laboratory confirmed cases	30,780	78,325	−61	−50% (−68%–−24%)†	−66% (−79%–−44%)†
		Slid positivity rate	11	22	−53	−28% (−41%–−11%)†	−37% (−51%–−20%)†
	**Nonmalaria**	Nonmalaria outpatient cases	1,663,624	1,170,565	42	−15% (−40%–21%)	−11% (−41%–34%)
		Nonmalaria inpatient cases	199,845	146,131	37	−12% (−41%–33%)	−8% (−43%–49%)
		Nonmalaria deaths	8,589	6,637	29	−22% (−52%–27%)	−16% (−53%–51%)

*A negative percentage (ratio between observed and predicted indicators level multiplied by 100) indicates a decrease se in the indicator in the year compared to baseline period*.

*A positive percentage indicate an increase of the indicator or impact in the year compared to baseline*.

† *: 95% Confidence Interval (CI) does not include zero and change of trend (pre versus post-intervention) is statistically significant*.

Concomitantly, non-malaria outpatient cases, inpatient cases, and deaths either increased or remained unchanged (Table 1, Figure 4a, 4c, 4d). The number of laboratory tests performed varied from a mean of 350,000 during 2001–2005 to 292,000 in 2011. The decrease in the number of microscopy tests performed is approximately similar to the decrease in the number of confirmed malaria cases (78,000 to 30,000) during the same time period.

A sensitivity analysis of the malaria cases for the 132 months covering the years 2001–2011 indicated that the month with the maximum change in the trendline was the 62^nd^ month (January 2006) for confirmed malaria and the 66^th^ month (June, 2006) for SPR, admissions and deaths 2006.

Among children under 5 years of age, observed inpatient malaria cases were 41% lower in 2011 than the mean of the observed values during 2001–2005 (Table 1), and lower by 81% (CI, 47%–94%) than the level predicted for 2011. In this age-group, malaria deaths were 73% (CI, 48%–86%) lower in 2011 than predicted by previous trends. Among those 5 years and older, malaria inpatient cases and malaria deaths were lower in 2011 than predicted based on 2001–2005 trends, however these changes were not statistically significant. The proportions of inpatient cases due to malaria (of all-cause inpatient) in children under 5 years fell from 23% in 2001–2005 to 11% in 2011; and malaria deaths fell from 17% to 8% of all-cause deaths during the same period. The proportion of inpatient cases due to malaria in 5 years and above fell from 15% to 6% and the proportion of malaria deaths decreased from 19% to 5%.

The observed number of confirmed malaria cases in 2011 was lower than the mean number of confirmed cases during 2001–2005 in all 10 regions (Table 2). The decrease in confirmed cases varied among regions from 36 to 99%. Similarly, the SPR and the number of malaria deaths were lower in all regions, while malaria inpatient cases were higher in 2011 in one region, Gambella. Confirmed malaria cases were lower in 2011 compared with 2001–2005 in 33 of 41 hospitals; inpatient cases were lower in 34 hospitals and malaria deaths were lower in 37 hospitals.

**Table 2:** Percentage change in inpatient malaria cases, malaria deaths and slide positivity rate in 2011 compared to pre-intervention period (2001–2005), by hospital and region, in 41 hospitals below 2000 m elevation, Ethiopia, 2001–2011.

Region	Hospital	Confirmed cases			SPR			Malaria admissions			Malaria deaths			% of population protected with LLIN	
		Mean (2001–2005)	2011	% Change in 2011	Mean (2001–2005)	2011	% Change in 2011	Mean (2001–2005)	2011	% Change in 2011	Mean (2001–2005)	2011	% Change in 2011	2005	2011
***Afar***	***Dubti***	***5089***	***2540***	***−50***	***39***	***26.8***	***−32***	***193***	***154***	***−20***	***27***	***8***	***−71***	***22***	***53***
Amhara	Dessie	975	89	*−*91	13	1.7	*−*87	254	11	*−*96	20	3	*−*86		
	Gondar Referal	483	399	*−*17	10	9.1	*−*8	286	313	9	34	25	*−*26		
	Woldia	455	77	*−*83	51	3.2	*−*94	134	45	*−*66	11	2	*−*83		
***Total Amhara***		*1914*	*565*	*−70*	*15*	*4.8*	*−68*	*674*	*369*	*−45*	*66*	*30*	*−55*	*10*	*48*
BGumuz	Asossa	5147	936	*−*82	41	41.5	2	559	334	*−*40	17	7	*−*58		
	Pawe	3341	1501	*−*55	28	28.0	0	2366	1382	*−*42	47	7	*−*85		
***Total Bgumuz***		***8489***	***2437***	***−71***	***35***	***32.0***	***−7***	***2924***	***1715***	***−41***	***64***	***14***	***−78***	***34***	***50***
***Dire Dawa***	***Dil Chora***	***742***	***24***	***−97***	***12***	***1.1***	***−91***	***1197***	***41***	***−97***	***87***	***4***	***−96***	***66***	***50***
***Gambella***	***Gambella***	***5167***	***3599***	***−30***	***31***	***21.4***	***−30***	***1045***	***2293***	***119***	***100***	***17***	***−83***	***95***	***100***
Harari	Hiwot Fana	624	6	*−*99	10	0.1	*−*99	497	22	*−*96	59	1	*−*99		
	Jegol	720	2	*−*100	13	0.2	*−*99	359	25	*−*93	38	3	*−*91		
***Total Harari***		***1343***	***8***	***−99***	***12***	***0.1***	***−99***	***855***	***47***	***−95***	***96***	***4***	***−96***	***62***	***70***
Oromia	Adama	NA	NA	NA	NA	NA	NA	120	121	0	13	4	*−*72		
	Ayira	1204	36	*−*97	9	0.6	*−*92	191	14	*−*93	18	2	*−*89		
	Bishoftu	NA	NA	NA	NA	NA	NA	221	52	*−*77	13	24	78		
	Bisidimo	686	0	*−*100	16	0.0	*−*100	169	0	*−*100	3	0	*−*100		
	Bule Hora	931	616	*−*34	46	8.4	*−*82	223	378	69	23	15	*−*36		
	Chiro	304	381	25	13	7.9	*−*40	272	95	*−*65	59	3	*−*95		
	Dembi Dolo	323	250	*−*23	13	3.6	*−*71	103	49	*−*53	15	5	*−*67		
	Gelemso	5195	6	*−*100	45	0.2	*−*100	4897	6	*−*100	86	0	*−*100		
	Gimbi Adventist	370	68	*−*82	10	1.6	*−*85	223	71	*−*68	10	0	*−*100		
	Ginnir	353	812	130	5	8.1	50	58	0	*−*100	3	0	*−*100		
	Jimma University	2820	1388	*−*51	19	8.9	*−*54	409	141	*−*66	58	19	*−*67		
	Mettu Karl	310	58	*−*81	5	0.9	*−*83	196	103	*−*48	25	7	*−*74		
	Mizan Aman	1740	503	*−*71	20	8.1	*−*60	581	309	*−*47	28	41	48		
	Nekemte	10363	298	*−*97	15	0.8	*−*95	1140	394	*−*65	63	15	*−*76		
	Shashemene	1076	90	*−*92	11	1.6	*−*86	361	92	*−*74	40	22	*−*45		
	St. Lukas Catholic	1287	667	*−*48	15	5.9	*−*60	443	571	29	30	46	53		
***Total Oromia***		***26963***	***5172***	***−81***	***17***	***4.1***	***−76***	***9608***	***2396***	***−75***	***488***	***201***	***−59***	***8***	***40***
SNNP	Arba Minch	5687	2962	*−*48	30	25.7	*−*15	3887	1952	*−*50	136	38	*−*72		
	Atat	3355	2097	*−*37	27	17.6	*−*36	1225	957	*−*22	100	29	*−*71		
	Butajira HC	1376	1757	28	22	12.9	*−*40	267	167	*−*37	14	21	51		
	Dilla	2558	3428	34	31	47.8	56	1333	449	*−*66	43	12	*−*71		
	Gebretsadik Showa	3697	163	*−*96	27	5.4	*−*80	21	46	115	0	0	*−*100		
	Jinka Zonal	2316	2097	*−*9	28	16.0	*−*43	689	726	5	40	22	*−*47		
	Tarecha	741	722	*−*3	31	11.0	*−*64	138	512	270	14	8	*−*42		
	Wolayita	1188	652	*−*45	21	8.8	*−*58	861	732	*−*15	59	40	*−*32		
	Yirgalem	1974	810	*−*59	25	6.3	*−*74	716	429	*−*40	156	50	*−*68		
***Total SNNP***		***22893***	***14687***	***−36***	***28***	***16.9***	***−41***	***9137***	***5969***	***−35***	***562***	***219***	***−61***	***26***	***41***
Somali	Gode	1114	554	*−*50	44	16.5	*−*62	246	166	*−*33	7	3	*−*63		
	Karamara Regional	35	5	*−*85	4	0.4	*−*90	41	15	*−*62	5	0	*−*100		
***Total Somalia***		***1149***	***559***	***−51***	***33***	***12.1***	***−63***	***287***	***181***	***−37***	***11***	***3***	***−78***	***17***	***45***
Tigray	Abi Adi	388	302	*−*22	29	8.3	*−*71	284	261	*−*8	10	2	*−*80		
	Adwa	2756	452	*−*84	38	14.7	*−*61	345	153	*−*56	6	2	*−*67		
	Alamata	623	251	*−*60	19	4.6	*−*76	1122	625	*−*44	31	4	*−*89		
	Suhul	957	773	*−*19	19	13.1	*−*29	680	217	*−*68	19	18	*−*8		
***Total Tigray***		***4724***	***1778***	***−62***	***27***	***9.8***	***−63***	***2430***	***1256***	***−48***	***66***	***25***	***−62***	***15***	***44***
***All hospital (regions)***		***78472***	***31370***	***−60***	***23***	***10.7***	***−53***	***28351***	***14421***	***−49***	***1568***	***525***	***−67***	***21***	***51***

*A negative percentage indicates a decrease of the indicator or impact in the year compared to baseline.*

### Seasonality

**Figure 5:**
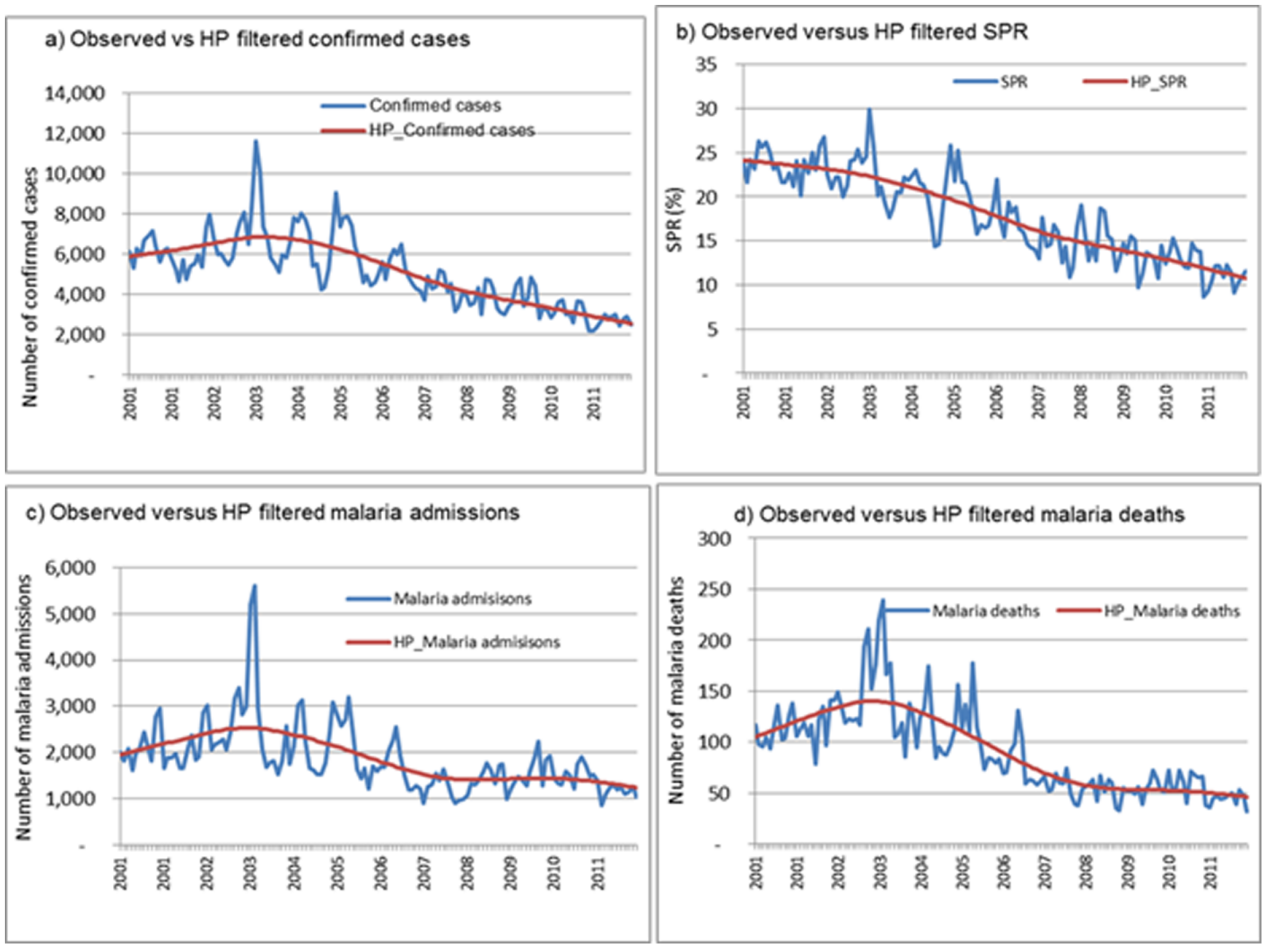
Time series of monthly confirmed malaria cases, slide positivity rate, admissions and deaths extracted by use of a Hodrick-Prescott filter with monthly smoothing parameter λ = 14,400 (HP  =  Hodrick-Prescott filter; MAl-IPDAll Ages  =  Inpatient malaria cases in all ages; Mal-DeathsAll Ages  =  Malaria deaths in all ages, SPRAll Ages  =  Slide positivity rate in all ages; PositiveAll ages  =  Number of confirmed cases).

Using the Hodrick-Prescott filter method to remove short-term (monthly) fluctuations and determine the long term time-series over multiple years, a decrease in all malaria indicators is seen (Figure 5). Confirmed malaria cases and SPR decreased through 2011, while malaria inpatient cases and malaria deaths decreased until 2008, followed by stability between 2008 and 2011. The spectral analysis showed that the fundamental frequency components of these series is one or annual.

**Figure 6:**
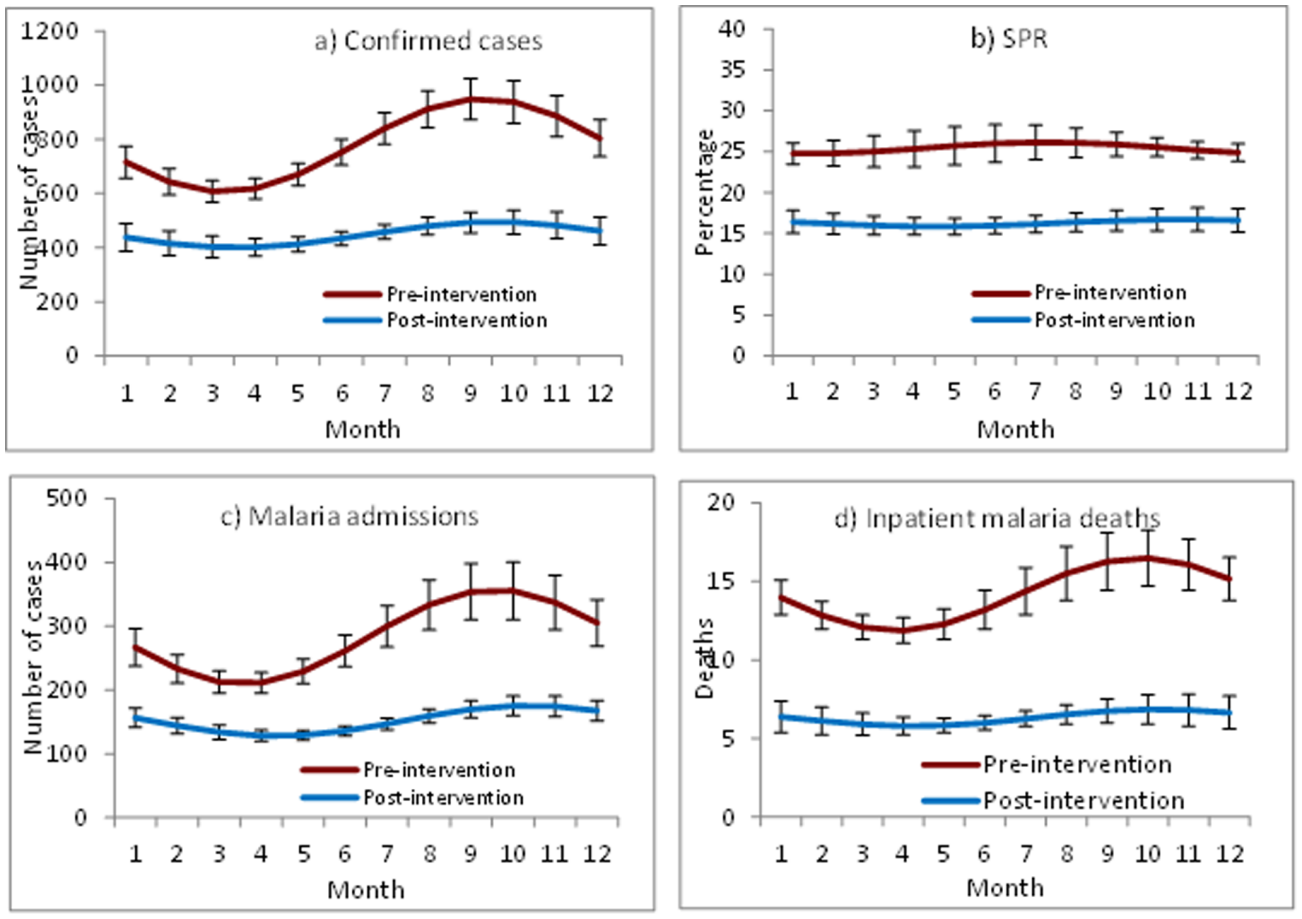
Monthly mean number of malaria confirmed cases, malaria inpatient cases, malaria deaths and slide positivity rate, 2001–2005 and 2006–2011, 41 hospitals, Ethiopia.

Monthly mean confirmed malaria cases, SPR, malaria inpatient cases, and malaria deaths were lower for all months during 2006–2011 (post-intervention period) compared to 2001–2005 (pre- intervention period (Figure 6 a, b, c, d). Malaria inpatient cases and malaria deaths varied widely by month during 2001–2005, while little variation in monthly means was seen during 2006–2011.

### Rainfall

**Figure 7:**
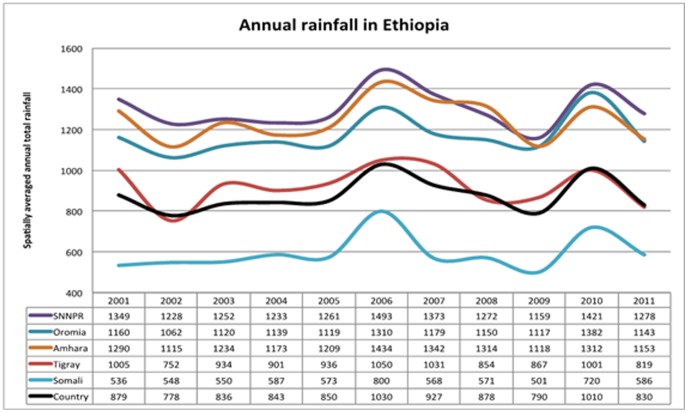
Trends of annually averaged rainfall (mm) in the major regions, 2001–2011, Ethiopia.

Precipitation data of five regions and national aggregates made available by the International Research Institute for Climate and Society (IRI) were analysed. No significant difference in precipitation between pre- and post-intervention years was observed (Figure 7). Rainfall remained favourable for malaria transmission and peaked in 2006 and 2010 while it was lowest in 2009 across the regions. A linear regression of rainfall on national aggregates of confirmed cases and SPR showed no significant effect (*p>0.05*). Linear regression on confirmed cases and SPR by region showed no significant effect during the pre-intervention period in all regions except in SNNP region (where rainfall had significant increasing effect on both). During the post-intervention period, rainfall had a significant increasing effect on confirmed cases and SPR in all the regions except in Oromia, where rainfall had significant decreasing effect (*p<05*) and in Tigray with no significant effect.

## Discussion

In this study reviewing data from hospitals in Ethiopia, malaria decreased between 2001 and 2011 by several measures. Among all age groups, the number of confirmed malaria cases, malaria inpatient cases and deaths declined by more than half, while malaria inpatient cases and malaria deaths among children under 5 years old decreased more than two-thirds from the numbers predicted if levels at the start of the decade had persisted. The observed declines in malaria cases and deaths were seen across all regions where malaria transmission occurs and could not be accounted for by changes in outpatient visits or by the number of diagnostic tests performed. During this time, measured rainfall was sufficient to support malaria transmission and levels of rainfall could not be consistently correlated with changes in malaria cases. Counts of cases and deaths were used in this analysis as the catchment population of each hospital was not known. Given the increase in population of Ethiopia during this time, the decline in malaria cases and deaths as rates per population may have been greater.

The downward trend in malaria cases and deaths was consistent across several approaches to the time series analysis. Comparing the observed number of cases in 2011 to the level predicted in 2011 by extrapolating trends from the first 5 years of the time period, 2001–2005, before full scale-up of malaria interventions, resulted in the largest measured decrease in cases and deaths. For confirmed cases, this can be explained in part by a slight increase in cases during the baseline period resulting in a higher predicted level in 2011 than the mean level during the 2001–2005. In contrast, for SPR, comparing the observed level in 2011 to the predicted level resulted in a smaller decrease due to an already decreasing trend of SPR in the baseline period. These examples demonstrate how characteristics of the baseline period chosen can affect the magnitude of measured changes over time [13]. Comparing the mid-point level of the regression line from 2006–2011 to the mean level 2001–2005 resulted in the smallest decrease for all primary indicators. Therefore this could be seen as the most conservative approach to this time series analysis. In addition to the decreasing trends seen in annual cases and deaths, lower numbers of malaria cases, admissions and deaths were seen when comparing the mean for each month during the post-intervention period to the same month in the pre-intervention period. Seasonal peaks also appeared lower in the post-intervention period compared to pre-intervention period.

The hospital data analysed for this study indicate that the decreases in cases and deaths coincided with the scale-up of malaria interventions. Analysis of the breakpoints in the time series of malaria cases and deaths were consistent with a change in the trend approximately when interventions were either introduced, or increasing during 2005 and 2006. The proportion of population potentially protected with LLINs increased from negligible levels prior to mass LLIN distribution starting in 2005 to levels 60% and above during 2006–2011. The proportion of the population protected by IRS also increased substantially during this time, reaching nearly 50% of the population at risk in 2008. By analysis of data on stock-outs at hospitals, ACTs were available in more than 90% of facilities after they were introduced in July 2004.

While confirmed malaria cases and the SPR decreased consistently until 2011, no additional decreases in malaria inpatient cases or malaria deaths were observed in recent years (during 2009–2011) compared to that of the early post-intervention period (2006–2008). This may be partly due to saturation of the protective effect of interventions following the attainment of the LLIN coverage and provision of ACTs by 2008. It may also be attributed to relatively slower changes in some of the major hospitals of high transmission areas in Oromia, SNNP and Gambella. A portion of LLIN distributed in 2006–2008 may also have been lost or rendered less effective during 2009–2011 due to reduced physical integrity and insecticidal waning after their estimated three-year effective lifespan. The measure of ACT availability used in this study, based on documentation of ACT stock-outs at facilities, does not provide a direct measure of what proportion of patients with malaria received adequate treatment, therefore it is unknown if changes in treatment patterns had an effect.

Assessment of trends in malaria cases and deaths and the effect of malaria control activities reported previously by Ethiopia, and other sub-Saharan African countries (Zanzibar [18], [19], Rwanda [20], The Gambia [21] and Sao Tome and Principe [22]) have utilized both facility data and household survey results and have shown significant decrease following scale up of interventions. Compared to the 2008 study in Ethiopia [23], the present study benefits from three additional years of data and attempts to address concerns regarding analysis of trends given large variability in malaria transmission based on historical data in Ethiopia and a short, possibly spurious baseline in available facility data.

This study is subject to several limitations. A key challenge in assessing the trends in malaria in Ethiopia is the historically high variability in baseline malaria transmission. An attempt was made to account for this in the analysis; however, the time period reviewed may have been too short to reveal longer term trends in malaria transmission that may have been unrelated to malaria interventions. Visiting hospitals and reviewing data at hospitals helped in minimizing reporting bias present in routine surveillance. Even so, excluding visited hospitals with incomplete data introduces its own potential bias; this is likely to be small as the excluded hospitals represent a small fraction of the hospitals visited. The main reason for the incomplete data at the hospitals was disappearances of record books and monthly reports (for some months or years particularly the early years) and improper handing over of records during change of health staff. The study did not include lower level health facilities and it is unclear if trends there would be the same as those seen in the hospitals considered. There were no data on the volume of patients cared for or the number of malaria cases identified through community case management in the Health Extension Programme. The effect of this program on trends in hospital malaria cases appears to be limited as the decline in malaria cases and deaths occurred two years before the scale-up of community case management in 2009 and, given health care seeking patterns in Ethiopia, only a third of patients treated in the community were likely to have been seen at hospitals [24]. In addition, significant decrease in malaria cases and deaths were observed against the background of increased all-cause outpatient and all-cause inpatient cases and deaths in the hospitals. Information on testing practices at the district hospitals was limited. The number of suspected cases presenting to health facilities that should be tested for malaria was not recorded in standard reporting forms and therefore, it was difficult to assess whether the proportion of suspect patients tested changed over time. The measures of malaria control intervention coverage in this study were crude estimates of these interventions in the community however, they did reflect the time period during which interventions were available and were adequate to denote large changes in intervention coverage. More precise measures of LLIN coverage and ACT treatment would be needed to more closely relate changes in coverage to changes in malaria occurrence. Given the very high ecological variations within a region, annual average rainfall for the major regions may not be conclusive to accurately assess the climatic effect on trends of malaria cases and deaths. In a separate analysis of rainfall data by the International Research Institute for Climate and Society, Columbia University, New York, USA, using Enhancing National Climate Services (ENACTS) (not shown here), for most regions there was lower rainfall in the pre-intervention (baseline) period (2000–2005) relative to the post-intervention period (2006–2010). In addition, analysis of temperature indicate that the climate of Ethiopia has been getting warmer over the last 30 years –particularly in highland areas. Higher temperatures and rainfall in the intervention period suggest that the climate suitability for malaria from 2006–2010 was above that of the baseline period. Any large-scale declines in malaria cases and deaths during 2006–2010 are unlikely therefore to be as a result of a drier or cooler climate and an associated reduction in transmission suitability.

The results of health facility-based changes in disease occurrences should be cautiously interpreted, as they may be influenced by changes in service utilization, testing rates, reporting, and other factors [25]. To the extent that these are addressed, review of hospital data from Ethiopia shows that malaria cases and deaths have decreased during the second half of the study period and these changes may be related to the increase in malaria control interventions. Given the history of malaria transmission in Ethiopia, strengthening of the surveillance system to allow regular monitoring of the disease trends is crucial to the success of the programme. Malaria in Ethiopia is highly heterogeneous and despite the recent reduction in malaria, the risk of resurgence and epidemics is still very real [5]. Intensive malaria control activities guided by epidemiological stratification with high resolution and adequate funding are therefore needed to reduce malaria transmission further and remove the risk of resurgence. In moderate-high transmission areas with considerable number of outpatient malaria cases, national malaria control programmes should consider surveillance on aggregates of outpatient cases and use line-listing of inpatient cases for assessing the use of interventions and causes of continued transmission in some areas. In low transmission areas where number of cases are manageable, line-listing of outpatient cases should be considered for in-depth geo-referencing and investigation of foci [26].

It has been difficult to discern trends in malaria cases and deaths from national aggregates reported in Ethiopia because of inconsistent and incomplete reporting [6]. This study was undertaken to attempt to account for reporting inconsistencies. Similar studies from sample hospitals will likely be required until the health information system becomes fully functional at national level to provide facility-level quality data to allow reliable assessment of trends of malaria cases and deaths; and other related indicators. Such studies would be stronger if complemented by trend analysis at levels of health centres and health posts to confirm if trends observed in hospitals are consistent with that of lower level facilities.
